# An Analytical Planning Model to Estimate the Optimal Density of Charging Stations for Electric Vehicles

**DOI:** 10.1371/journal.pone.0141307

**Published:** 2015-11-17

**Authors:** Yongjun Ahn, Hwasoo Yeo

**Affiliations:** Department of Civil and Environmental Engineering, Korea Advanced Institute of Science and Technology (KAIST), Daejeon, Republic of Korea; Lanzhou university of Technology, CHINA

## Abstract

The charging infrastructure location problem is becoming more significant due to the extensive adoption of electric vehicles. Efficient charging station planning can solve deeply rooted problems, such as driving-range anxiety and the stagnation of new electric vehicle consumers. In the initial stage of introducing electric vehicles, the allocation of charging stations is difficult to determine due to the uncertainty of candidate sites and unidentified charging demands, which are determined by diverse variables. This paper introduces the Estimating the Required Density of EV Charging (ERDEC) stations model, which is an analytical approach to estimating the optimal density of charging stations for certain urban areas, which are subsequently aggregated to city level planning. The optimal charging station’s density is derived to minimize the total cost. A numerical study is conducted to obtain the correlations among the various parameters in the proposed model, such as regional parameters, technological parameters and coefficient factors. To investigate the effect of technological advances, the corresponding changes in the optimal density and total cost are also examined by various combinations of technological parameters. Daejeon city in South Korea is selected for the case study to examine the applicability of the model to real-world problems. With real taxi trajectory data, the optimal density map of charging stations is generated. These results can provide the optimal number of chargers for driving without driving-range anxiety. In the initial planning phase of installing charging infrastructure, the proposed model can be applied to a relatively extensive area to encourage the usage of electric vehicles, especially areas that lack information, such as exact candidate sites for charging stations and other data related with electric vehicles. The methods and results of this paper can serve as a planning guideline to facilitate the extensive adoption of electric vehicles.

## Introduction

To mitigate the impact of climate change and to maximize the sustainability of our society, numerous governments encourage auto industries to develop alternative transportation systems to replace conventional modes of transportation. The electric vehicle (EV) is a promising alternative because it can solve many environmental problems by reducing carbon emissions, which contribute to the reduction of air pollution in urban areas. EVs have become increasingly beneficial from an environmental perspective and an economical perspective compared with conventional vehicles [1]. Governmental investments, regulations and incentives are being developed to encourage the adoption of EVs [2, 3]. Numerous major auto manufacturers demonstrate interests in EVs and have developed passenger and commercial vehicles [4].

Despite these advantages, significant barriers against the extensive adoption of EVs exist; they currently represent a small penetration of markets in EV service [5]. The most frequent concerns about EVs are the limited driving range, charging station availability, and high vehicle purchase costs [6]. The range anxiety caused by limited driving range and the low availability of charging stations discourage the acceptance by new consumers and restrain the economic benefits of EVs. For instance, the early adopters of EVs may use their vehicles for only short trips and drive fewer average miles than their travel experience without range anxiety [7]. The efficient allocation of charging stations for EVs is important to the adoption of the EV system because it is expected to resolve concerns about limited driving range and charging station availability. Another critical issue for adopting an EV is the charging process. The charging process requires a greater amount of time than the time required for gasoline or diesel fueling systems. Charging may require several hours, even with a fast charging system [8]. For efficient allocation of charging stations for an EV, the delay time in a queue caused by the longer charging time should be considered. To encourage the usage of EVs for a low-emission society, well-organized charging infrastructure planning that considers range anxiety and delay time by charging queue is required.

The problem related to charging infrastructure is critical to the development of an EV system [5]. The lack of charging infrastructure can interrupt the adoption of EVs, which may reduce many incentives for investing charging infrastructures. Although charging stations have been constructed in many cities, only few studies have determined the optimal locations of charging stations for maximizing electricity-based travel [8]. Some recent studies have attempted to determine where to establish charging stations to reduce the range anxiety and to improve the recharging efficiency. Shukla et al. determined optimal locations based on the maximum impact on the number of recharged EVs [9]. Wang’s model has been performed for determining the minimum recharge time and length of stay at each location [10]. The objective of Nie and Ghamani’s’s model is to determine the battery size and charging capacity with minimal social costs [11]. Various mathematical models have been proposed to determine optimal locations for refueling, charging and battery swapping stations. Nichlolas et al. proposed a geographic information system (GIS)-based model that employs the p-median [12]. Lin et al. developed the fuel-travel-back approach, which employs the distribution of vehicle miles traveled (VMT) [13]. Wang and Lin’s model is a variant path-based set covering model [14]. Kubi et al. proposed a flow refueling location problem (FRLP) [15]. Dong et al. utilized an activity-based assessment method to determine an optimal location for siting charging stations [7]. Pan et al. developed a stochastic model to determine the best location for battery swapping stations in a vehicle to power grid [16]. Jung et al. proposed an itinerary interception location problem that applies to an EV taxi [17]. In these previous studies, traffic flow volume data [18], the distribution of existing petrol refueling stations data [19] and vehicles ownership data [20] were employed to estimate the charging demand. The simulation of trips based on origin-destination (OD) pairs have been conducted to determine the charging demand [17, 21, 22]. Real-world trip data were also employed for analyzing the charging demand; however, sample data for private vehicles are limited [7, 8].

Galus et al. proposed an integrated method comprising vehicles technology, transportation simulation, and power system [23]. This method can assess the impacts of electric mobility on those three domains in future technology advances. Bayram et al. presented the method of allocating a network of fast charging station, which is minimizing the cost function of charging capacity, speed of charging and vehicles arrival rate [24]. Yudovina et al. considered a decentralized routing design and pricing strategies through social optimal function with little to no queueing [25]. They also incorporated technological constraints and heterogeneity of charging preferences into the problem. Another social optimal function framework based on driving distance and queueing delays is proposed in Hung et al. [26]. Wirges et al. proposed a dynamic spatial model of charging infrastructure, which consists of sub-models simulating EV ownership, charging demand and allocation of charging station [27]. Liu et al, proposed the mathematical model minimizing the total costs of investment cost, maintenance cost, operation costs, and network loss costs in the planning period [28]. Chen et al. presented the cost function minimizing EV users’ station access costs with the consideration of parking demand [29]. An optimal design framework for battery charging/swap station based on life cycle cost (LCC) is presented in Zheng et al [30].

As the use of EVs has gradually increased, various meaningful studies have been conducted to analyze the charging location problem, charging demand, and optimal planning. Various aspects of optimizing the performance of charging station infrastructure have been examined in the literature recently. However, the aspect of deriving the optimal density of charging stations has rarely been studied. In this study, we introduce an analytical approach to estimating the optimal density of charging stations with consideration of previous studies. The objective of this study is to provide the approximate optimal density of charging stations for wide areas, which are subsequently aggregated to conduct city-level planning. In the initial planning phase of introducing EVs, decision makers such as local governments who installing charging infrastructures, may gain insight from the results in the deployment planning phase. A detailed deployment solution, such as determining the exact locations for stations and setting a diverse number of chargers at each station is beyond the scope of this paper. The results of this paper help establish a detailed deployment plan. The method and results of this paper can present an approximate solution for efficiently allocating charging stations and provide a planning guideline for governments, even when no reference data of real EV travel patterns and exact candidate locations of charging stations. The following sections describe a model to estimate the required density of EV charging stations, and a numerical study and case study are conducted based on the proposed model.

## Estimating the Required Density of EV Charging Stations (ERDEC) Model

### ERDEC scheme

**Fig 1:**
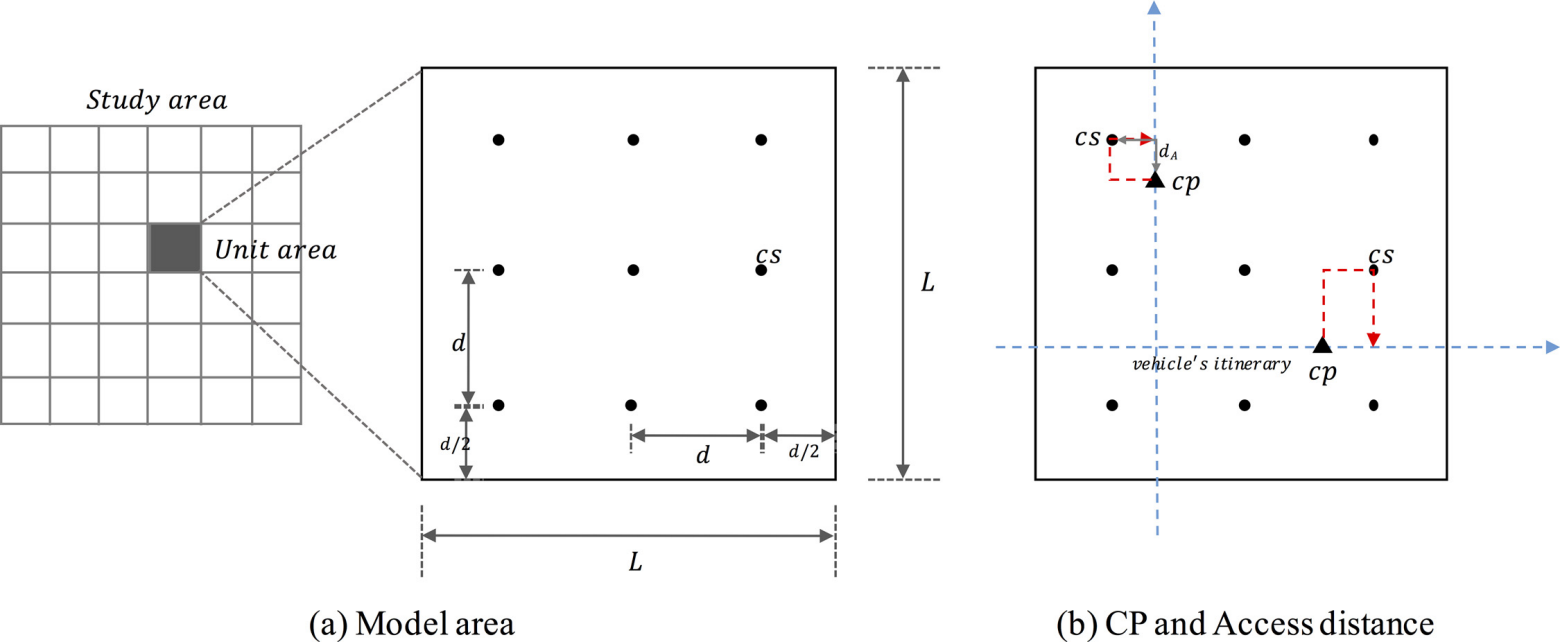
ERDEC scheme.

To introduce Estimating the Required Density of EV Charging stations (ERDEC) model, we consider a simplified situation for a given unit square area. The side length of this unit area is *L*; thus, the area size is *L* × *L*, as illustrated in Fig 1. The charging stations (CSs), which are expressed as black dots in Fig 1(A), are assumed to be evenly distributed with equivalent spacing. Let *d* be the distance between two CSs. The density of CSs can be expressed by *L* and *d*.

The objective of this model is to determine the optimal density that yields minimal cost. The cost is composed of several items, such as the access cost of CSs, the delay time cost of charging, charger installation costs and the operating cost of CSs. To calculate these costs, we form the following assumptions: (1) the vehicles randomly pass a given area in a vertical or horizontal direction; (2) each CS has same number of chargers; (3) when a charging request event occurs (based on the state of charge (SOC) level), the EVs should go to the nearest CS. (4) CSs are public, and fast charging systems are adopted in all CSs. A detailed description of each assumption is demonstrated in the following section.

The first and second assumptions reflect homogeneous environments. The first assumption is assumed because the layout of the street network is a grid type network in an urban area. We assumed that the random distribution exhibits a uniform probability distribution. The third assumption implies that the charging points (CPs) generate a vehicle’s itinerary interception, as illustrated in Fig 1(B). We assumed that a charging event occurs whenever the SOC level is less than 15% of the charging level because the warning light for the shortage of battery is activated at that level. Although drivers do not always charge at 15% of the SOC level, they recognize the need to charge and seek charging stations. For the fourth assumption, although the majority of EVs tend to use home chargers (slow charging system) due to the lack of public charging infrastructures, this study considers drivers who do not develop anxiety regarding an EV driving range. Similar to conventional gas stations, public CSs and fast charger systems should be installed.

### Mathematical formulation

The objective of this subsection is to define the related parameters of the ERDEC model and to derive an equation by expressing density.

#### The number of charging stations (CSs)

The number of CSs is denoted by *N*_*cs*_. Let *δ* be the density of CSs, which is the number of CSs over the unit area size, as expressed by the following equation:

$$\delta =\frac{N_{cs}}{Area\;size}=\frac{N_{cs}}{L^{2}}$$

Thus, the number of CSs is defined by the following form:

$$N_{cs}=\delta L^{2}$$ (1)

#### Additional access distance

Let *d*_*A*_ be the access distance from CP to CS, where *d*_*A*_ ∈ (0, *d*), as illustrated in Fig 1(B). Therefore, the expectation of *d*_*A*_ is defined as *E(d*_*A*_ *)* = $\frac{d}{2}$. Let $l_{EV_{dis}}$ denote the available distance after charging. In this case, *d*_*A*_ should be less than 0.15 $\times l_{EV_{dis}}$ because the charging events occur at a level below 15% of the SOC level, that is, the constraint of *d*_*A*_.

$$E(d_{A})=\frac{d}{2}for\;d_{A}\leq 0.15l_{EV_{dis}}$$

where *$l_{EV_{dis}}$* is the available distance after charging.

The number of CSs on one side is $\frac{L}{d}$; thus, the total number of CSs in the area is ${\left(\frac{L}{d}\right)}^{2}$.

Then, we have

$$N_{cs}={\left(\frac{L}{d}\right)}^{2}$$ (2)

From Eqs (1) and (2), the expectation of *d*_*A*_ is

$$E(d_{A})=\frac{1}{2\sqrt{\delta }}$$

**Fig 2:**
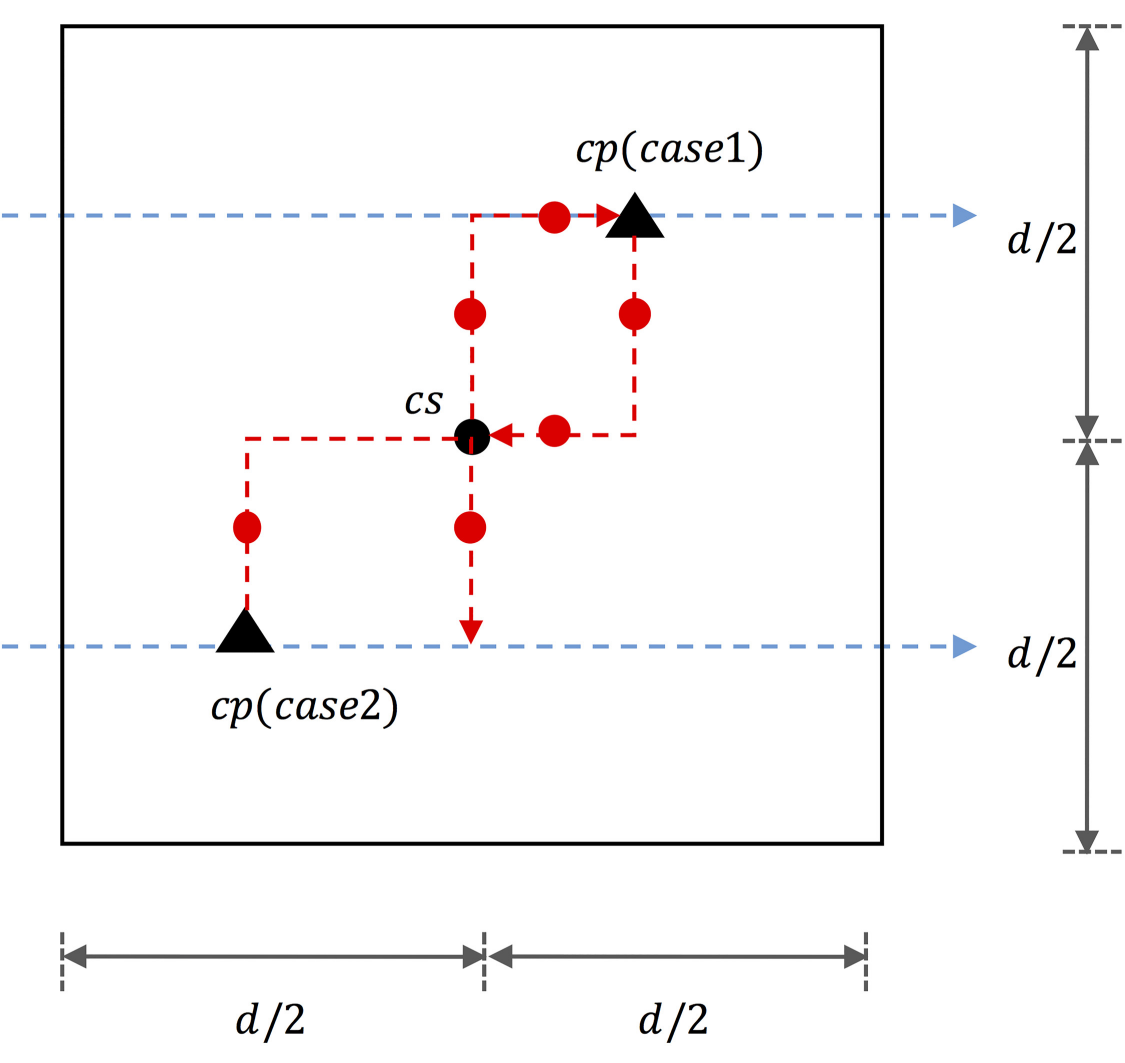
Additional trip for charging.

The additional trip (additional access distance) is divided into two cases regardless of whether the nearest charging station is located in the travelling direction. Compared with the original trip, the original trips are denoted by red lines with by red dots, as illustrated in Fig 2.

Case 1) opposite direction

In the case that the nearest charging station is located on the opposite direction of an itinerary, *E(Additional trip_case1)* is *d*.

Case 2) travelling direction

In the case that the nearest charging station is located in the same direction of an itinerary, *E(Additional trip_case2)* is $\frac{d}{2}$.

Thus, we obtain

$$E\left(Additional\;trip\right)=\frac{3}{4}d=\frac{3}{4\sqrt{\delta }}$$ (3)

#### The number of CP

The number of CPs is denoted as *N*_*cp*_ and is directly associated with the charging demands. To estimate the charging demand, we need to quantify the number of vehicles required by charging. Let *N*_*veh*_ be the number of passing electric vehicles per T hour, where T is the time state. The number of charging vehicles, which is the number of CPs (*N*_*cp*_), is calculated subject to the length of the area *L* and the available distance after charging, $l_{EV_{dis}}$ [available distance in this model is $0.85l_{EV_{dis}}$ because charging events occur below 15% of the SOC level]. When a vehicle passes the *L* length of distance with $0.85l_{EV_{dis}}$ limited driving range, the required number of charging points in a cell is $\left(\frac{L}{{0.85l}_{EV_{dis}}}\right)$. For example, if *L* is $0.85l_{EV_{dis}}$, the vehicles passing the cell should have a one-time charging event, regardless of the SOC level at the time of entering the cell. If *L* is less than $0.85l_{EV_{dis}}$, the expected number of charging points is stochastically calculated as $\left(\frac{L}{{0.85l}_{EV_{dis}}}\right)$. Then, the total number of charging points (number of charging vehicles) is defined by the following equation:

$$N_{cp}=N_{c\_veh}=\frac{LN_{veh}}{0.85l_{EV_{dis}}}$$ (4)

#### Delay time in queue for charging

**Fig 3:**
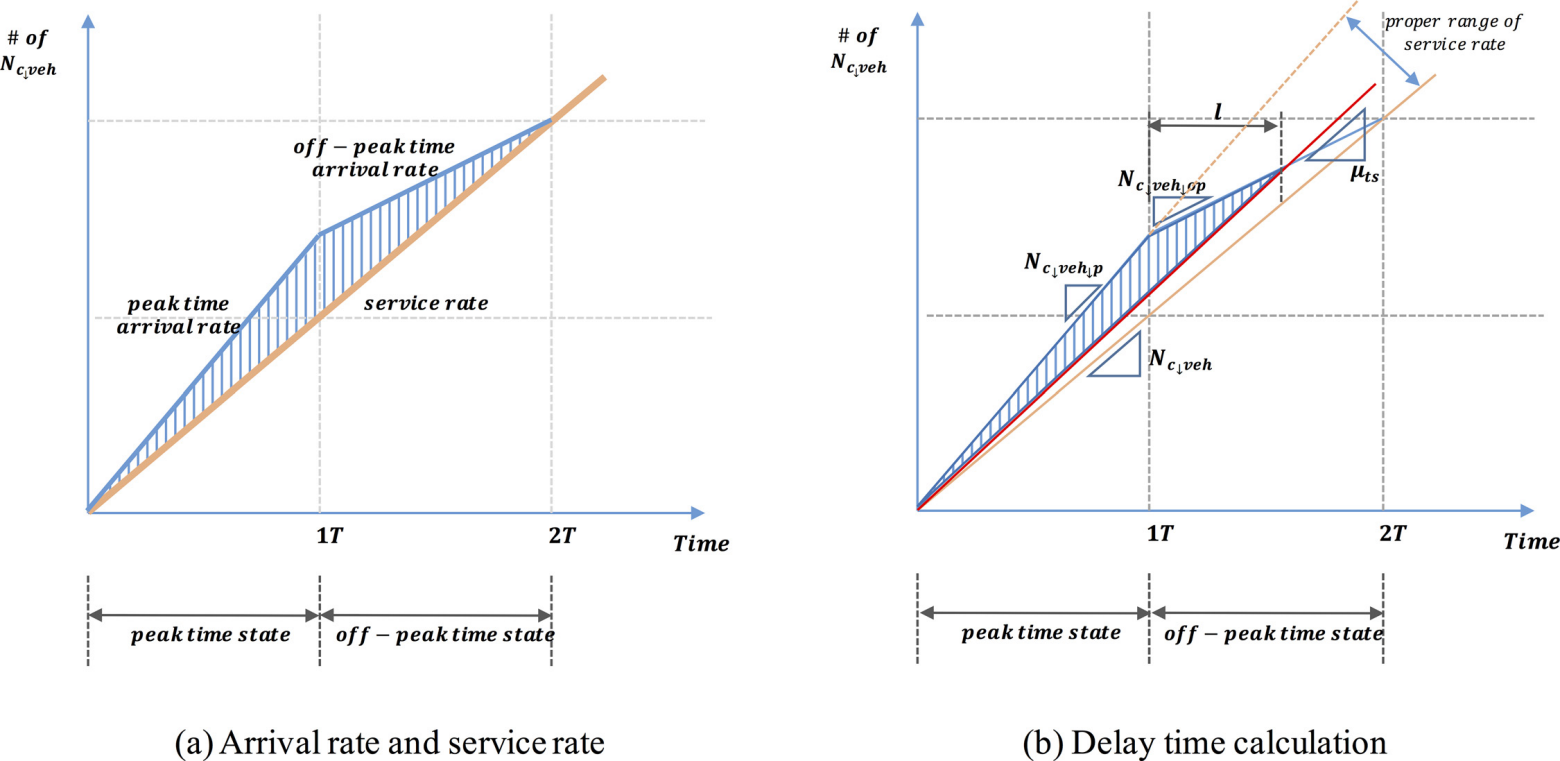
Delay time.

A critical obstacle to EV adoption is the extended charging time. Thus, the delay time caused by a charging queue is considered to be an important part for estimating the density of CSs. If we install a sufficient number of CSs to match the peak time charging demand, no delay time for charging would be incurred. However, this case may cause a waste of budget and public resources during off-peak time periods. To obtain an optimal solution, we should consider both the peak time and off-peak time demands. Therefore, we consider two states (peak time state and off-peak time state) in this study by assuming that the delay disappears as the state changes from peak time to off-peak time. The triangular area size formed by the peak time arrival rate, off-peak time arrival rate and service rate represents the delay time, as illustrated in Fig 3(A).

Let *T*_*f_ch*_ denote the charger’s full charging time. The service rate of a charger is $\frac{1}{T_{f\_ch}}$, and the constraint indicates that *T*_*f_ch*_ is less than 1T. Then, the total service rate (*μ*_*ts*_) in this area is expressed by the following equation:

$$\mu_{ts}=\frac{kN_{cs}}{T_{f\_ch}}=\frac{k\delta L^{2}}{T_{f\_ch}},\;for\;T_{f\_ch}\leq \;1T$$ (5)

where *k* is the number of chargers at each CS.

Because the delay should disappear in 2T (two states), we assume that the total number of charging vehicles during peak time and off-peak time is the equivalent to the number of charging vehicles during 2T hours, as expressed by the following equation

$$TN_{c\_veh\_p}+TN_{c\_veh\_op}=2TN_{c\_veh}$$ (6)

where *N*_*c_veh_p*_ is the number of charging vehicles during peak times and *N*_*c_veh_op*_ is the number of charging vehicle during off-peak times.

Let *p* be the proportion of peak time demand. In this case, the proportion of off-peak time demand is (1 –*p*). The range of *p* extends from 0.5 to 1 because the peak time demand must exceed the off-peak time.

$$p\;:\left(1-p\right)=N_{c\_veh\_p}:N_{c\_veh\_op}$$ (7)

where *p* is the proportion of peak time demand 0.5 ≤ *p* ≤ 1.

From Eqs (6) and (7), we derive

$$N_{c\_veh\_p}=2pN_{c\_veh}$$ (8-1)

$$N_{c\_veh\_op}=2\left(1-p\right)N_{c\_veh}$$ (8-2)

If *μ*_*ts*_ < *N*_*c_veh*_, the delay does not disappear within 2T (two states) due to the lack of CSs. No additional delay time is incurred when *μ*_*ts*_ > *N*_*c_veh_p*_ due to the overestimation of CSs. Thus, the proper range of service rate is *N*_*c_veh*_ ≤ *μ*_*ts*_ ≤ *N*_*c_veh_p*_, as illustrated in Fig 3(B), which becomes the constraint of delay time equation.

As illustrated in Fig 3(B), the area size of the triangle that represents the delay time is calculated as

$$area\;size=\left(1T+l\right)\times \frac{T\left(N_{c\_veh\_p}-\mu_{ts}\right)}{2}$$

where

$$l=\frac{T\left(N_{c\_veh\_p}-\mu_{ts}\right)}{\left(\mu_{ts}-N_{c\_veh\_op}\right)}$$ (9)

From Eqs (8-1), (8-2) and (9), the triangular size is calculated as follows:

$$area\;size=\left\{\frac{T^{2}N_{c\_veh}\left(2p-1\right)\left(2pN_{c\_veh}-\mu_{ts}\right)}{\left(\mu_{ts}-2\left(1-p\right)N_{c\_veh}\right)}\right\}$$ (10)

Then, the total delay time in the queue for charging is expressed with Eqs (4), (6) and (10) as the following functional form

$$delay\;time=\left\{\frac{T^{2}LN_{veh}\left(2p-1\right)\left(2pLN_{veh}T_{f_{ch}}-0.85l_{EV_{dis}}k\delta L^{2}\right)}{0.85l_{EV_{dis}}\left(0.85l_{EV_{dis}}k\delta L^{2}-2\left(1-p\right)LN_{veh}T_{f_{ch}}\right)}\right\}$$ (11)

where *μ*_*ts*_ ≥ *N*_*c_veh*_; if *μ*_*ts*_ > *N*_*c_veh_p*_, the delay time is zero.

### Cost model of ERDEC

Because the objective of the ERDEC model is to obtain the optimal density that yields the lowest cost, the total cost should be calculated. Assume that the access cost from CP to CS is *Cost*_*A*_, the delay cost in the charging queue is *Cost*_*D*_, the fixed installation cost of the chargers is *Cost*_*I*_, and the operating cost of CSs is *Cost*_*O*_. Minimizing the sum of *Cost*_*A*_, *Cost*_*D*_, *Cost*_*I*_ and *Cost*_*O*_ is employed as the objective function of the ERDEC model.

$$min(Total\;Cost)=Cost_{A}+Cost_{D}+Cost_{I}+Cost_{O}$$

The detailed expression of the total cost is as follows:

$$Total\;cost=\left[c_{1}\times total\;additional\;trip+c_{2}\times total\;delay\;time+c_{3}\times the\;total\;number\;of\;chargers+c_{4}\times the\;total\;number\;of\;stations\right]$$

where

*c*_1_ = value of distance per km
*c*_2_ = value of time *per hour*
*c*_3_ = charger installation cost *per hour*
*c*_4_ = operating cost of charging station *per hour*

The time period that calculated the total cost reflects 2T hour due to the delay time. The methods for calculating each cost are described as follows:

#### Cost_A_: [c_1_ × total additional trip]

*Cost*_*A*_ is the total additional trip cost. It can be expressed as the multiplication of the total additional trips and the value of distance. The total additional trip from a CP to a CS is expressed by Eq (3) as follows:

$$\underset{i\in 2TN_{c_{veh}}}{\sum }additional\;trip_{i}=E\left(Addtional\;trip\right)\times 2TN_{c_{veh}}$$

From Eqs (3) and (4), the total additional trip is

$$Total\;additional\;trip=\frac{3TLN_{veh}}{1.7\sqrt{\delta }l_{EV_{dis}}}$$

constraint:

$$d\leq \;0.15l_{EV_{dis}}$$

This constraint is derived by the expression of density as follows:

$$\delta \geq \frac{1}{{\left(0.15l_{EV_{dis}}\right)}^{2}}$$

We obtain

$$Cost_{A}=c_{1}\left(\frac{3TLN_{veh}}{1.7l_{EV_{dis}}\sqrt{\delta }}\right)$$ (12)

constraint:

$$\delta \geq \frac{1}{{\left(0.15l_{EV_{dis}}\right)}^{2}}$$

#### Cost_D_: [c_2_ × total delay time]

*Cost*_*D*_ is the total delay time cost in the queue for charging. It can be expressed as the multiplication of the total delay time and the value of time. The total delay time has been derived in the previous section (Eq (11)). If *μ*_*ts*_ > *N*_*c_veh_p*_, the delay time is always zero. According to Eqs (5) and (8-1), if $\delta >\frac{2pN_{veh}T_{f\_ch}}{0.85kLl_{EV_{dis}}}$, the delay time is zero.

$$Total\;delay\;time=\left\{ \begin{array}{l} \left(\frac{T^{2}LN_{veh}\left(2p\;\text{-}\;1\right)\left(2pLN_{veh}T_{f_{ch}}\;\text{-}\;0.85l_{EV_{dis}}k\delta L^{2}\right)}{0.85l_{EV_{dis}}\left(0.85l_{EV_{dis}}k\delta L^{2}\;\text{-}\;2\left(1\;\text{-}\;p\right)LN_{veh}T_{f_{ch}}\right)}\right),\;if\;\delta \leq \frac{2pN_{veh}T_{f\_ch}}{0.85kLl_{EV_{dis}}}\\ \;0\;,\;if\;\delta >\frac{2pN_{veh}T_{f\_ch}}{0.85kLl_{EV_{dis}}} \end{array}\right.$$

constraint:

$$\delta \geq \frac{N_{veh}T_{f\_ch}}{0.85kLl_{EV_{dis}}}$$

This constraint is derived from *μ*_*ts*_ ≥ *N*_*c_veh*_, which is the constraint of the Eq (11).

Therefore, *Cost*_*D*_ is

$$Cost_{D}=\left\{ \begin{array}{l} c_{2}\left(\frac{T^{2}LN_{veh}\left(2p\;\text{-}\;1\right)\left(2pLN_{veh}T_{f_{ch}}\;\text{-}\;0.85l_{EV_{dis}}k\delta L^{2}\right)}{0.85l_{EV_{dis}}\left(0.85l_{EV_{dis}}k\delta L^{2}\;\text{-}\;2\left(1\;\text{-}\;p\right)LN_{veh}T_{f_{ch}}\right)}\right),\;if\;\delta \leq \frac{2pN_{veh}T_{f\_ch}}{0.85kLl_{EV_{dis}}}\\ \;0\;,\;if\;\delta >\frac{2pN_{veh}T_{f\_ch}}{0.85kLl_{EV_{dis}}} \end{array}\right.$$ (13)

constraint:

$$\delta \geq \frac{N_{veh}T_{f\_ch}}{0.85kLl_{EV_{dis}}}$$

#### Cost_I_: [c_3_ × the total number of chargers]

*Cost*_*I*_ is the total installation cost of the chargers. It can be expressed as the multiplication of the total number of chargers and the installation cost of the chargers. The total number of chargers is *kN*_*cs*_, where *k* is the number of chargers at each CS. Thus, using Eq (1), the total number of chargers is expressed as follows:

$$The\;total\;number\;of\;chargers=kN_{cs}=k\delta L^{2}$$

Then, we obtain

$$Cost_{I}=c_{3}2Tk\delta L^{2}$$ (14)

#### Cost_O_: [c_4_ × the total number of stations]

*Cost*_*O*_ is the total operating cost of CSs. It can be expressed as the multiplication of total number of CSs and the operating cost of a CS. The total number of CSs is defined by *N*_*cs*_, which is denoted as *δL* ^2^ in Eq (1).

Therefore,

$$Cost_{O}=c_{4}2T\delta L^{2}$$ (15)

### The objective function of ERDEC

#### The objective function and constraint

The total cost (*TC*) can be written based on the previous subsection (Eqs (12), (13), (14) and (15)) as

$$TC=\left\{ \begin{array}{l} c_{1}\left(\frac{3TLN_{veh}}{1.7l_{EV_{dis}}\sqrt{\delta }}\right)+c_{2}\left(\frac{T^{2}LN_{veh}\left(2p\;\text{-}\;1\right)\left(2pLN_{veh}T_{f_{ch}}\;\text{-}\;0.85l_{EV_{dis}}k\delta L^{2}\right)}{0.85l_{EV_{dis}}\left(0.85l_{EV_{dis}}k\delta L^{2}\;\text{-}\;2\left(1\;\text{-}\;p\right)LN_{veh}T_{f_{ch}}\right)}\right)+c_{3}2Tk\delta L^{2}+c_{4}2T\delta L^{2},\;if\;\delta \leq \frac{2pN_{veh}T_{f\_ch}}{0.85kLl_{EV_{dis}}}\\ c_{1}\left(\frac{3TLN_{veh}}{1.7l_{EV_{dis}}\sqrt{\delta }}\right)+c_{3}2Tk\delta L^{2}+c_{4}2T\delta L^{2},\;if\;\delta >\frac{2pN_{veh}T_{f\_ch}}{0.85kLl_{EV_{dis}}} \end{array}\right.$$

where

*c*_1_ = value of distance per km
*c*_2_ = value of time *per hour*
*c*_3_ = charger installation cost *per hour*
*c*_4_ = operating cost of charging station *per hour*
*L* = *one side length of the area*
*N*_*veh*_ = *the number of passing electric vehicles per T hour*
*p* = *proportion of peak time demand*, 0.5 ≤ *p* ≤ 1
*k* = *the number of chargers at each charging station*
*T*_*f_ch*_ = *full charging time*, *T*_*f_ch*_ ≤ 1T
$l_{EV_{dis}}$ = *available EV mileage after charging*
*T* = *time state*

constraint:

$$\delta \geq max\left(\frac{1}{{\left(0.15l_{EV_{dis}}\right)}^{2}},\;\frac{N_{veh}T_{f\_ch}}{0.85kLl_{EV_{dis}}}\right)$$

#### The first derivative

The decision variable of *TC* is *δ*. The first derivatives of *TC* respect to *δ* is as follows:

$$\frac{\partial TC}{\partial \delta }=\left\{ \begin{array}{l} c_{3}2TkL^{2}+c_{4}2TL^{2}-c_{1}\left(\frac{3TLN_{veh}}{3.4\delta^{3/2}l_{EV_{dis}}}\right)-c_{2}\left(\frac{kL^{3}\left(2p-1\right)T^{2}N_{veh}}{0.85kL^{2}\delta l_{EV_{dis}}-2L\left(1-p\right)N_{veh}T_{f\_ch}}\right)-c_{2}\left(\frac{kL^{3}\left(2p-1\right)T^{2}N_{veh}\left(2LpN_{veh}T_{f\_ch}-0.85kL^{2}\delta l_{EV_{dis}}\right)}{{\left(0.85kL^{2}\delta l_{EV_{dis}}-2L\left(1-p\right)N_{veh}T_{f\_ch}\right)}^{2}}\right),\;if\;\delta \leq \frac{2pN_{veh}T_{f\_ch}}{0.85kLl_{EV_{dis}}}\\ c_{3}2TkL^{2}+c_{4}2TL^{2}-c_{1}\left(\frac{3TLN_{veh}}{3.4\delta^{3/2}l_{EV_{dis}}}\right),\;if\;\delta >\frac{2pN_{veh}T_{f\_ch}}{0.85kLl_{EV_{dis}}} \end{array}\right.$$

Constraint is $\delta \geq max\left(\frac{1}{{\left(0.15l_{EV_{dis}}\right)}^{2}},\;\frac{N_{veh}T_{f\_ch}}{0.85kLl_{EV_{dis}}}\right)$.

With this differential equation, we can obtain the optimal density. In the next section, the numerical results of applying the objective function and differential equation of ERDEC are presented.

## Numerical Study Based on ERDEC Model

### Data description

To demonstrate the effect of the ERDEC model, specific numerical information, such as EV driving history or charging time data, is required. The values of these parameters can be flexible according to the locational characteristics and vehicle type. This study employs real EV taxi operation data in Daejeon city, South Korea, which were collected in September 2013 (Daejeon Techno Park, 2014). Although these data are gathered from EV taxis, they provide information about EV consumption efficiency, available driving range after charging, and charging time. Table 1 is the average maximum running distance collected from the EV taxi operation experiments. The experimental data are gathered from three EV taxis in September, during which the average temperature is 21.4°C. The EV taxi model is Renault SM3 Z.E with a battery that has a capacity of 22 kwh. The average maximum running distance is 143.5 km, and the average distance per power consumption is 6.52 km/kWh. The average charging time of EV taxis from 15% of the SOC level to 100% of the SOC level is approximately one hour in September.

**Table 1:** Experimental data of real EV taxi.

September 2013	Travel distance (km/day)	Electricity consumption (kWh)	Consumption efficiency (km/kWh)	Available range (km)
**1** ^**st**^ **week**	85.84	12.66	6.78	149.2
**2** ^**nd**^	138.92	21.22	6.55	144.1
**3** ^**rd**^	97.70	15.24	6.41	141.0
**4** ^**th**^	133.14	20.97	6.35	139.7
***Average***	***113*.*90***	***17*.*52***	***6*.*52***	***143*.*5***

Lee and Choi [31] estimated the value of travel time. They determined that the average value of time for passenger vehicles is 8,604 won in 2011. Considering the average rate of inflation (2.5%) in three years, the average value of time in 2014 is 9,266 won.

The installation cost of a fast charger may include not only the cost of the charger material and the cost of labor but also the cost of the electric transformer and the cost of pulling high-voltage power lines because grid and transformer upgrades are required if installations are not performed at high voltage gird levels. The total cost of a fast charger installation is approximately 26,700,000 won/ea [32]. The operating cost of CSs can vary depending on additional facilities. Because the labor cost is the most expensive part of the CS operating costs, it is a vital component for analyzing the effect of the ERDEC model. Thus, the labor cost is primarily considered as the CS operating cost in this study. The labor cost, which is 5,210 won, is calculated by the minimum wage in Korea in 2014 [33]. Table 2 summarizes these costs, which serve as the coefficient values of the objective function.

**Table 2:** Compilation of information about EV driving and charging.

Coefficient factors	Cost	Remarks (EV)
***c***_**1**_	16.9 won/km	1 kWh = 110 won, 6.52 km/kWh
***c***_**2**_	9,266 won/hr	
***c***_**3**_	508 won/hr	26,700,000 won/ea (assumed life cycle is 6 years)
***c***_**4**_	5,210 won/hr	Minimum wage of labor in 2014

The other parameters of the ERDEC model are listed in Table 3. These values serve as the baselines of the model. As previously mentioned, we set the parameters *T*_*f_ch*_ and $l_{EV_{dis}}$ to be 1 hour and 143.5 km, respectively, because they are technological parameters. Other parameters include the regional parameter values, which are arbitrarily defined as the baseline of the model.

**Table 3:** Parameters as the baseline of model.

Parameter		Description	Value
Technological	*T*_*f_ch*_	*full charging time*	1 hr
	$l_{EV_{dis}}$	*available EV distance*	143.5 km
Regional	*L*	*one side length of the area*	1 km
	*N*_*veh*_	*the number of passing EV per T hr*	1000
	*p*	*proportion of peak time demand*	0.6
	*k*	*the number of chargers at station*	1
	*T*	*time state*	1

**Fig 4:**
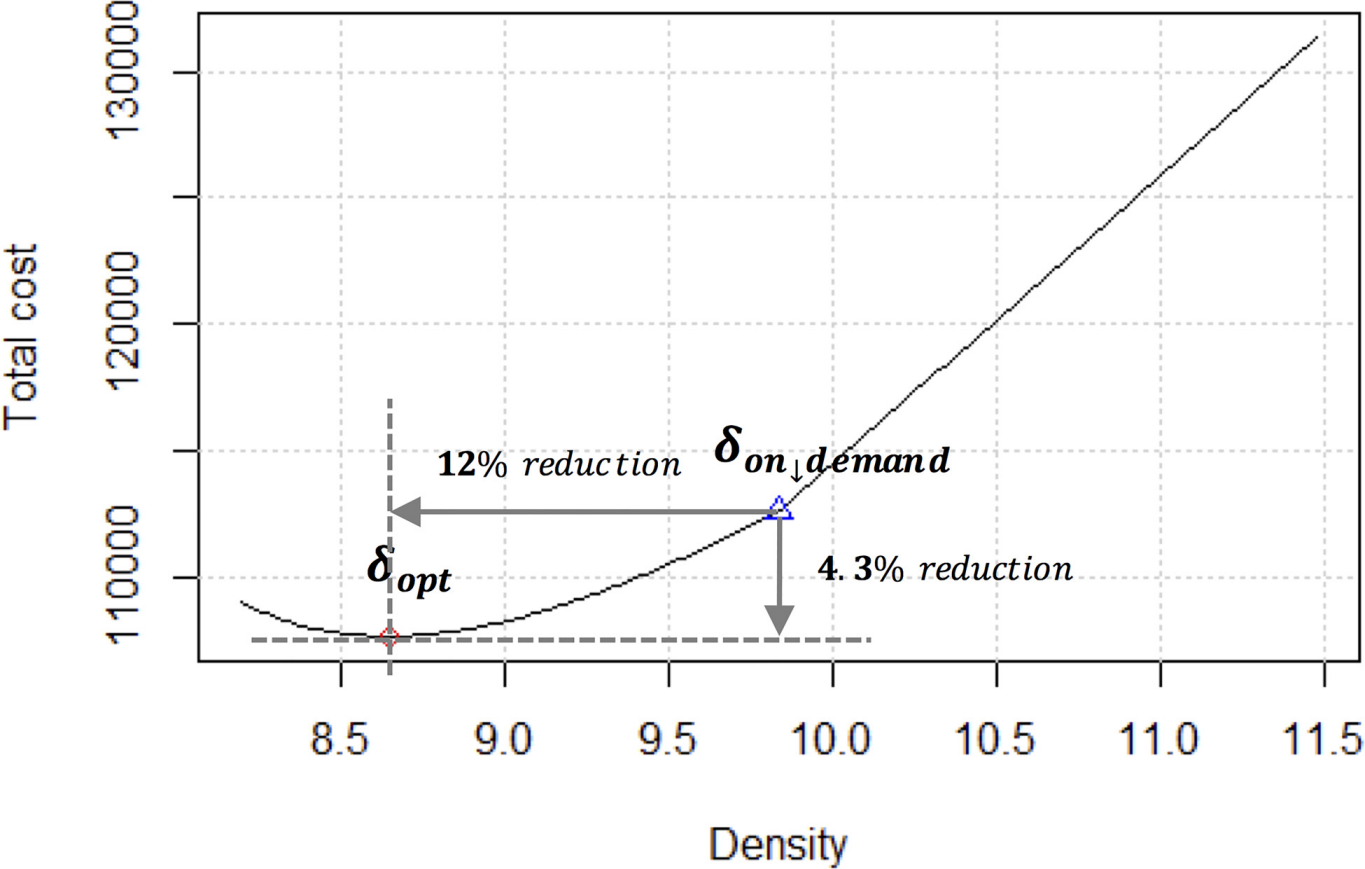
Plot of ERDEC model (baseline).

Given these parameters, as depicted in Fig 4, TC shows that the optimal solution (*δ*_*opt*_) is 8.65, where the optimal total cost is 107,623 won. The triangular point (*δ*_*on_demand*_) in Fig 4 represents the density value when the supply of the CSs matches the demand of all charging vehicles. At this point, *δ*_*on_demand*_ is 9.83 and the total cost is 112,542 won. If the CSs are installed according to the charging demands, public resources may be wasted. The proposed ERDEC model in this study demonstrates a 12% density reduction and a 4.3% cost reduction in the baseline case.

### The effect of regional parameters

To comprehensively analyze the results of different conditions, we compare the results in multiple conditions by changing the regional parameter values. Because these parameters reflect the regional characteristics, the objective of this subsection is to describe the correlation according to the variations in the parameters instead of calculating the proper values of the parameters.

#### The length of area: L

**Fig 5:**
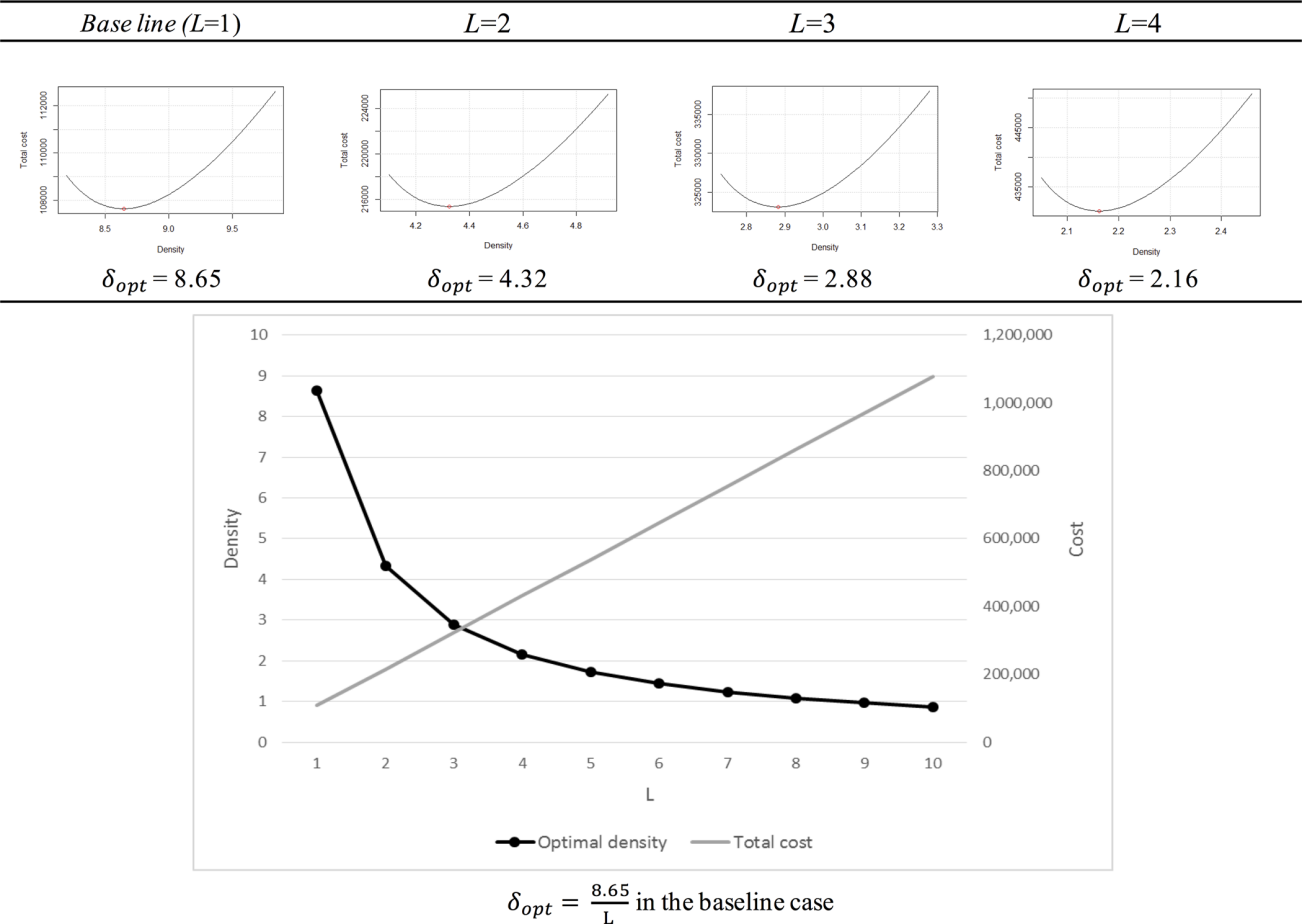
The variation in *L*.

Parameter *L* is the searching radius for estimating the density of CSs according to charging demands. When the number of charging demands remains constant, a larger *L* value has a lower density value but requires a higher total cost, as plotted in Fig 5.

The proper length of *L* for estimating density is dependent on the study sites and the number of passing vehicles. The parameter *L* is determined based on the characteristics of a study area, such as traffic volume, land use, and administrative district.

#### The number of passing EVs: N_veh_

**Fig 6:**
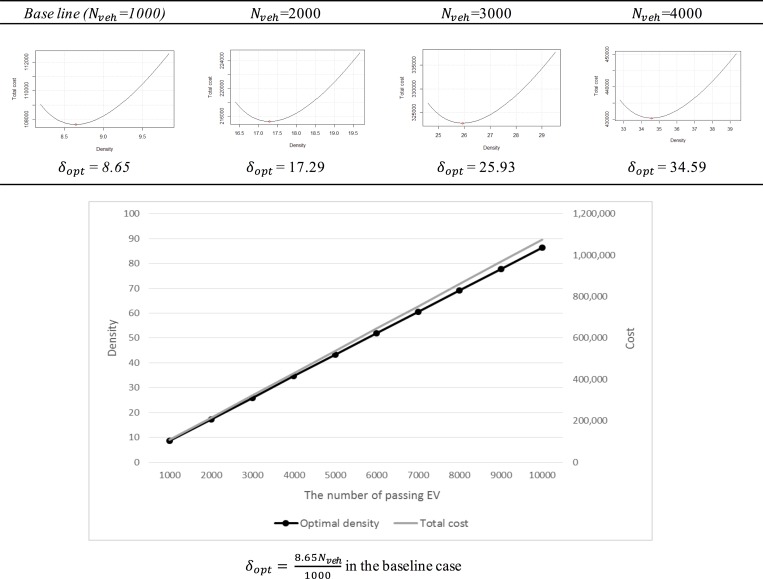
The variation in *N*_*veh*_.

Parameter *N*_*veh*_ is the most important value for the ERDEC model because it is the input value that directly reflects the charging demand. This value can be classified by the current traffic flow pattern on a study site or by the predicted number of future EVs. The optimal density and the total linear cost increase as the number of passing EVs increases, as shown in Fig 6.

#### The proportion of peak time demand: p

**Fig 7:**
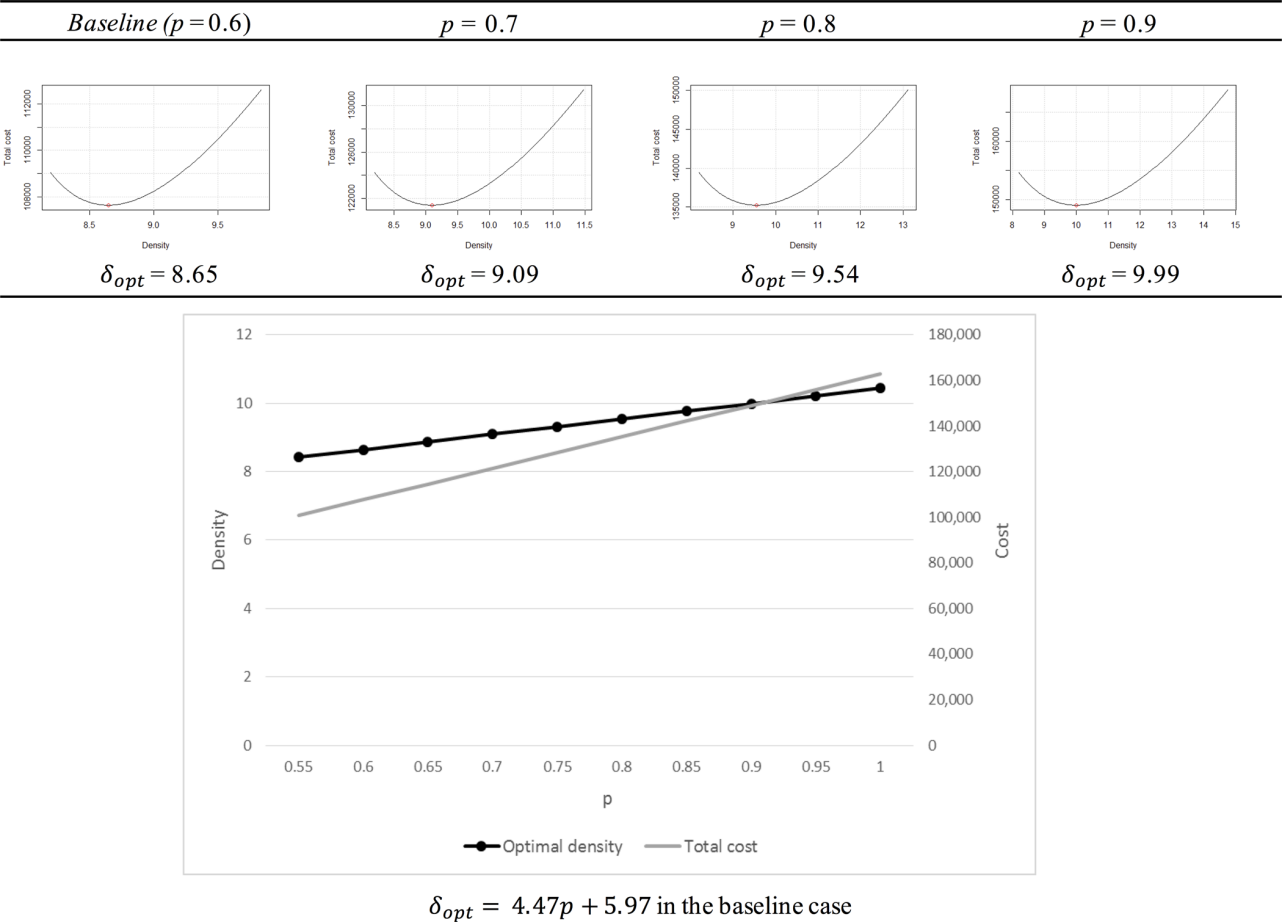
The variation in p.

Parameter *p* denotes the proportion of peak time demand, which reflects the ratio of peak time and off-peak time demands during the time period 2T. A higher *p* value reflects a significant difference between the peak time demand and off-peak time demand; thus, the total delay time increases. Fig 7 describes the plot of the optimal density and the total cost according to the variation in *p*. The optimal density and total cost have a positive correlation with *p*.

#### The number of chargers at each charging station: k

**Fig 8:**
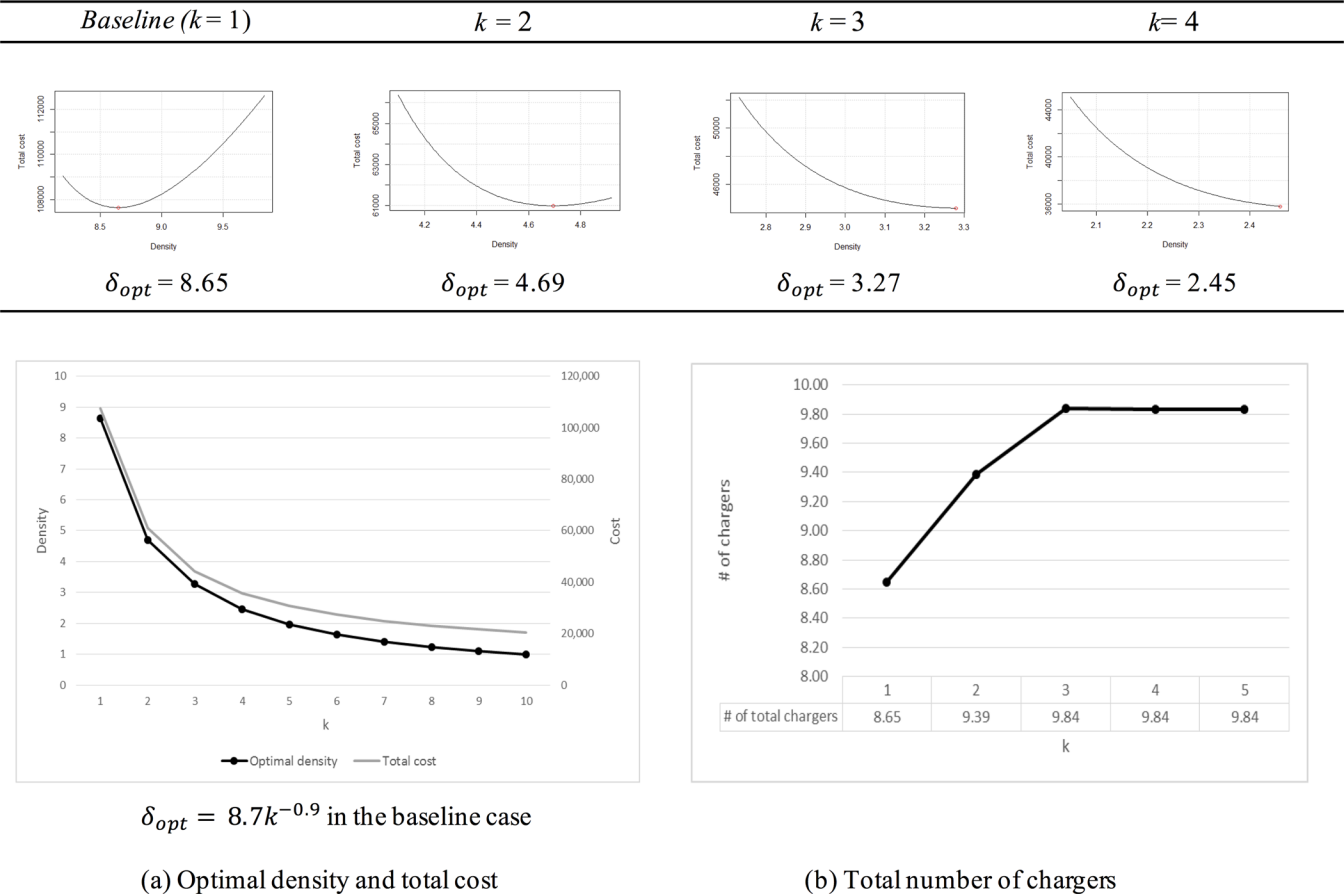
The variation in k.

Because the ERDEC model is a simplified model for approximately estimating the density for homogeneous conditions in the unit cell, the number of chargers cannot be dynamically adjusted for each station. Although the *k* value is equivalent for the same cell, the effect of *k* variations can be analyzed. Because the operating cost of a CS is more expensive than the installation cost of a charger, the number of chargers at each CS, *k*, shows a negative correlation with the optimal density and the total cost, as illustrated in Fig 8. To minimize the total cost and increase efficiency, additional chargers should be installed at one CS if no constraints exist, such as location size for installation and the maximum number of chargers at each station. In the baseline case, in which *L* is 1 km and *N*_*veh*_ is 1000, the optimal density has the same value as the on_demand density (the right side end point of plot is on_demand density) if *k* is greater than three, as illustrated in Fig 8. In Fig 8(B), the total number of chargers (9.84) remains constant when *k* exceeds three. To minimize the total number of chargers, a *k* value of 1 or 2 is appropriate in the baseline case. The ERDEC model is suitable because a small number of chargers tend to be installed in many locations at the initial phase of introducing an EV. The proper value of *k* can be selected based on regional characteristics.

### The effect of technological parameters

Problems with range anxiety and long charging times can be addressed by future technological development. In this section, changes in the optimal density and total cost are examined by the variation in the technological parameters. The quantitative benefits of technological improvements can be predicted with the proposed model.

#### The full charging time: T_f_ch_

**Fig 9:**
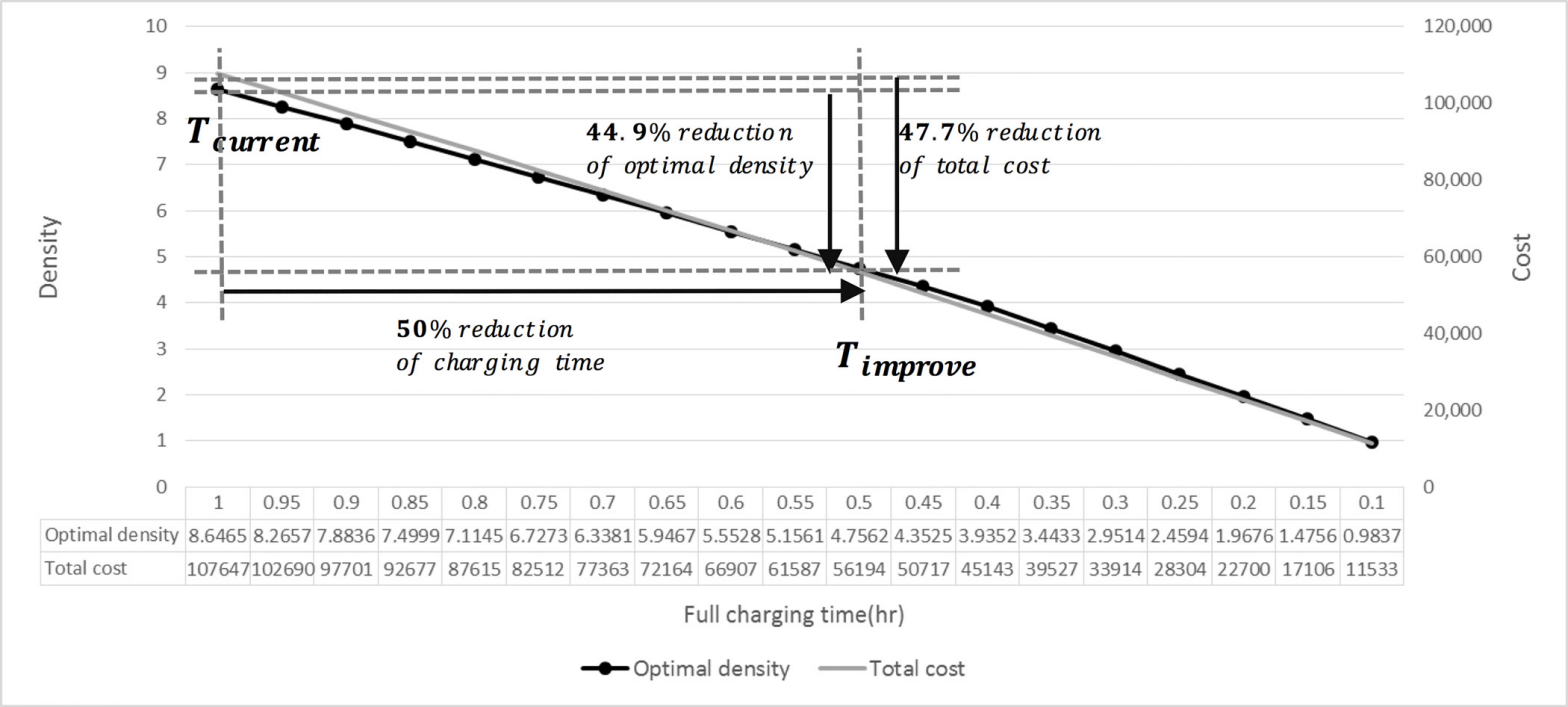
The variation of *T*_*f_ch*_.

Technological advancements in battery and charger systems may reduce the required charging time. Fig 9 indicates that both the optimal density and the total cost decrease with a reduction in charging time.

If the charging time decreases to one half of the current time duration (from 1 hr to 0.5 hr) by technological improvements, the optimal density would decrease by 44.9% and the total cost would be reduced by 47.7%.

#### Available distance after charging: $l_{EV_{dis}}$

**Fig 10:**
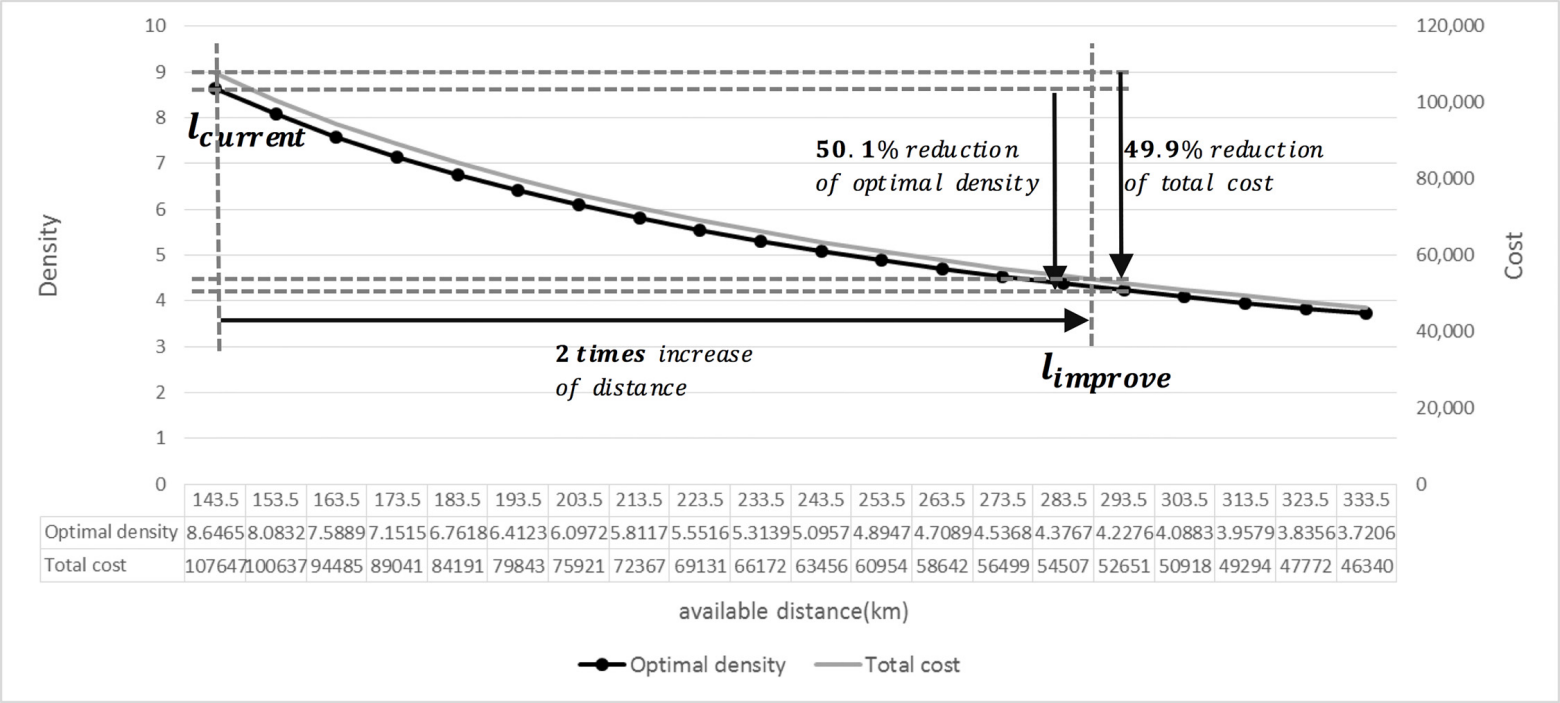
The variation in $l_{EV_{dis}}$.

Another significant benefit from future advancements in battery-related technologies is an increased EV distance after charging. As $l_{EV_{dis}}$ gradually increases, the optimal density and total cost decrease, as illustrated in Fig 10.

If the available distance after charging ($l_{EV_{dis}}$) increases by twice the current distance (from 143.5 km to 287 km) by technological improvements, the optimal density would decrease by 50.1% and the total cost would be reduced by 49.9%; When $l_{EV_{dis}}$ is 287 km, the optimal density is 4.32 and the total cost is 53843 won.

### The effect of coefficient factors

**Fig 11:**
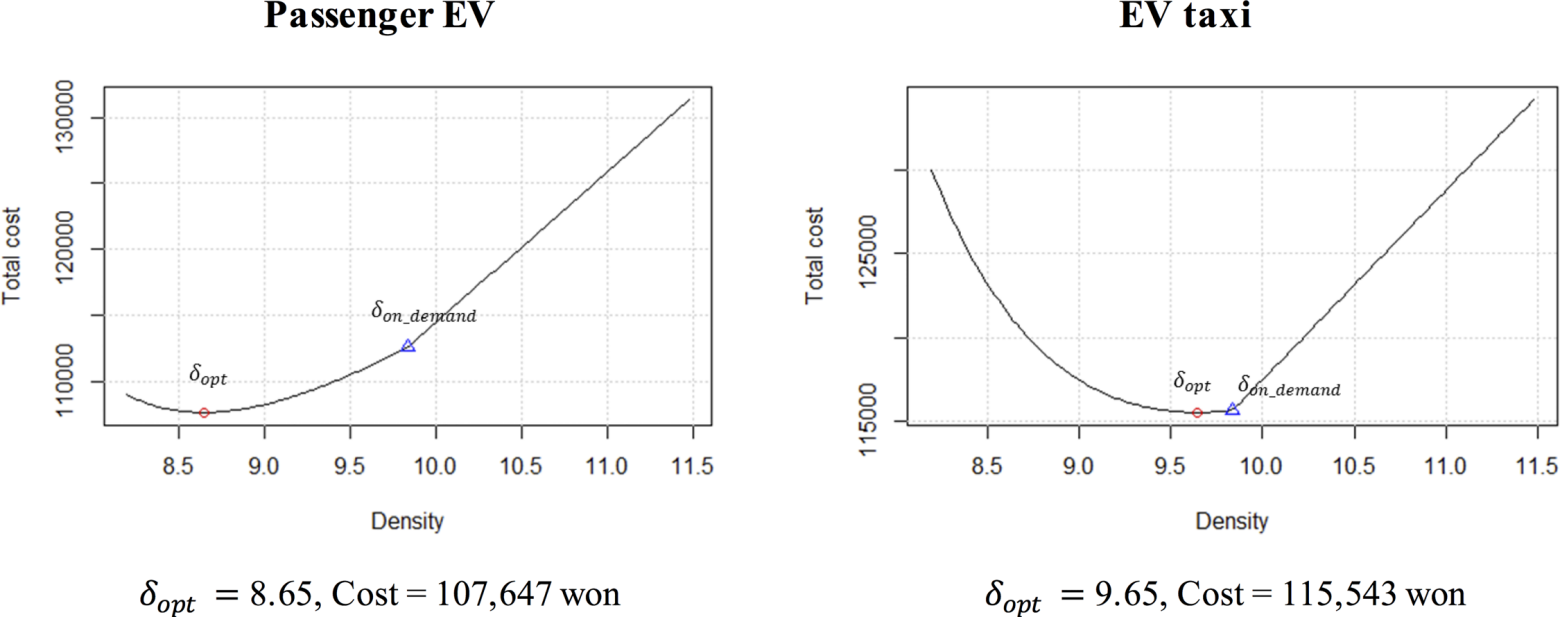
Comparison between passenger EV and EV taxi.

In the previous subsections, the variations in the regional and technological parameters are analyzed. This subsection examines the effect of coefficient factors. Table 2 lists the coefficient factors of general EVs. No significant differences in the coefficient values were observed for the same country. This study uses taxi data to conduct a comparison with other situations. The values of distance and time for taxis are greater than the values of distance and time for other auto vehicles, as shown in Table 4. These values are extracted from the Daejeon city report for taxi operation cost [34]. In the case of the baseline parameter values for EV taxis, the optimal density (*δ*_*opt*_) is 9.65 and the total cost at this density point is 115,543 won. The analysis with Fig 11 indicates that the difference between the optimal density and the density on demand for EV taxis is less than the difference between the optimal density and the density on demand for general EVs. This finding reflects that the installation of additional CSs for EV taxis is required because the value of time for taxis is much higher than the value of time for general auto vehicles.

**Table 4:** Coefficient factors of EV taxis.

Coefficient factors	Cost	Remarks (EV taxi)
***c***_**1**_	604.5 won/km	revenue/day: 200,002 won, distance/day: 330.89 km
***c***_**2**_	20,000 won/hr	operating time/day: 10 hr
***c***_**3**_	508 won/hr	same as passenger EV
***c***_**4**_	5,210 won/hr	same as passenger EV

## Case Study: Application to the Real World

**Fig 12:**
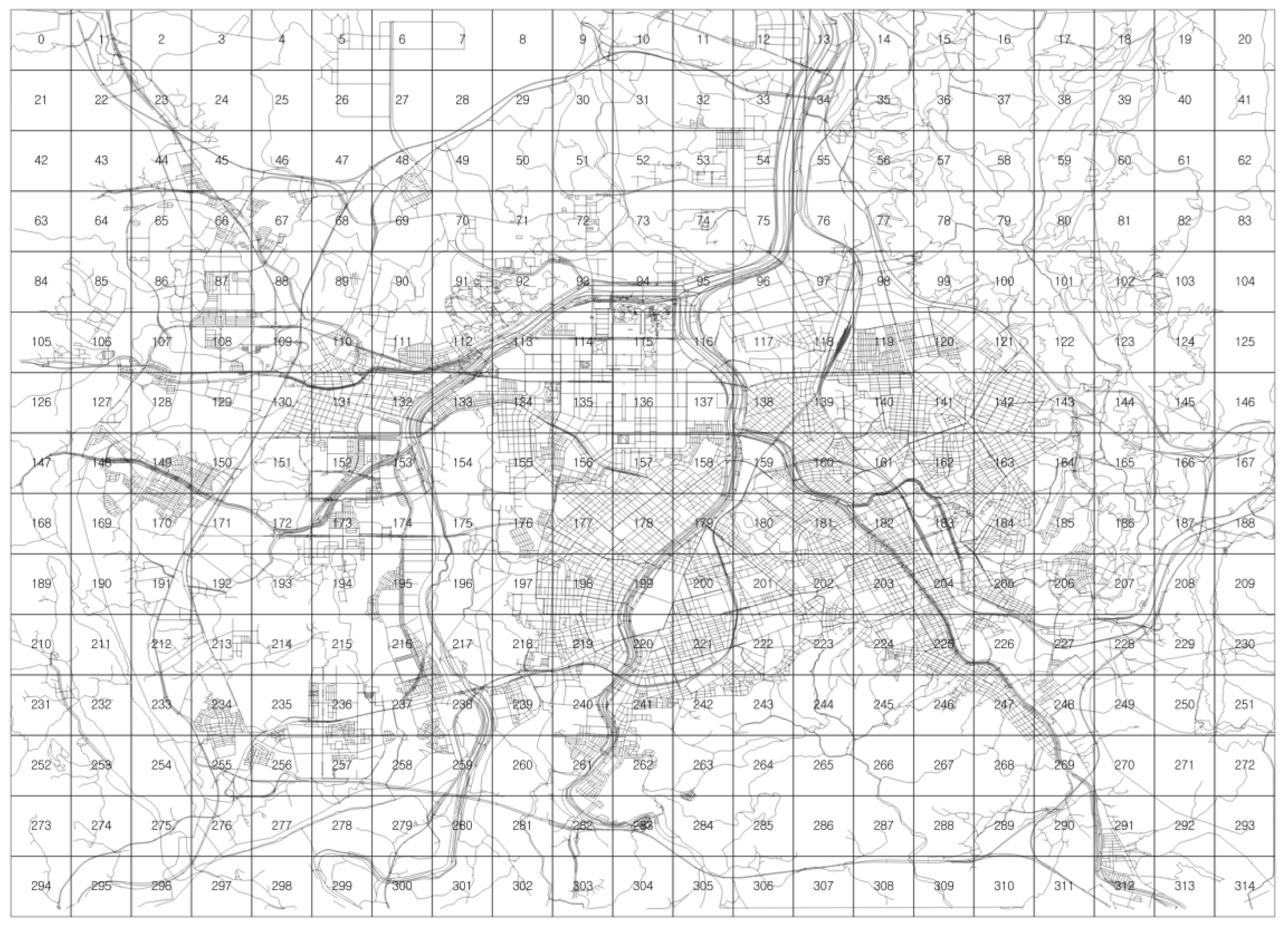
Daejeon area divided by 1 km×1 km cells.

To demonstrate the application of the ERDEC model to the real world, Daejeon city, South Korea is selected as an experimental study area. Daejeon city covers an area of 539.97 km^2^ with a population greater than 1,550,000. Daejeon has attempted to transform to a low-carbon city via numerous transportation programs, including incentives for electric vehicle use. Thus, Daejeon needs an initial plan for the efficient deployment of charging infrastructures. Fig 12 presents the Daejeon area divided by 1 km × 1 km squares, which is employed as the base unit area in the ERDEC model. The total number of squares is 315. In this area, the ERDEC provides an approximate analytical solution for planning the distribution of CSs.

**Fig 13:**
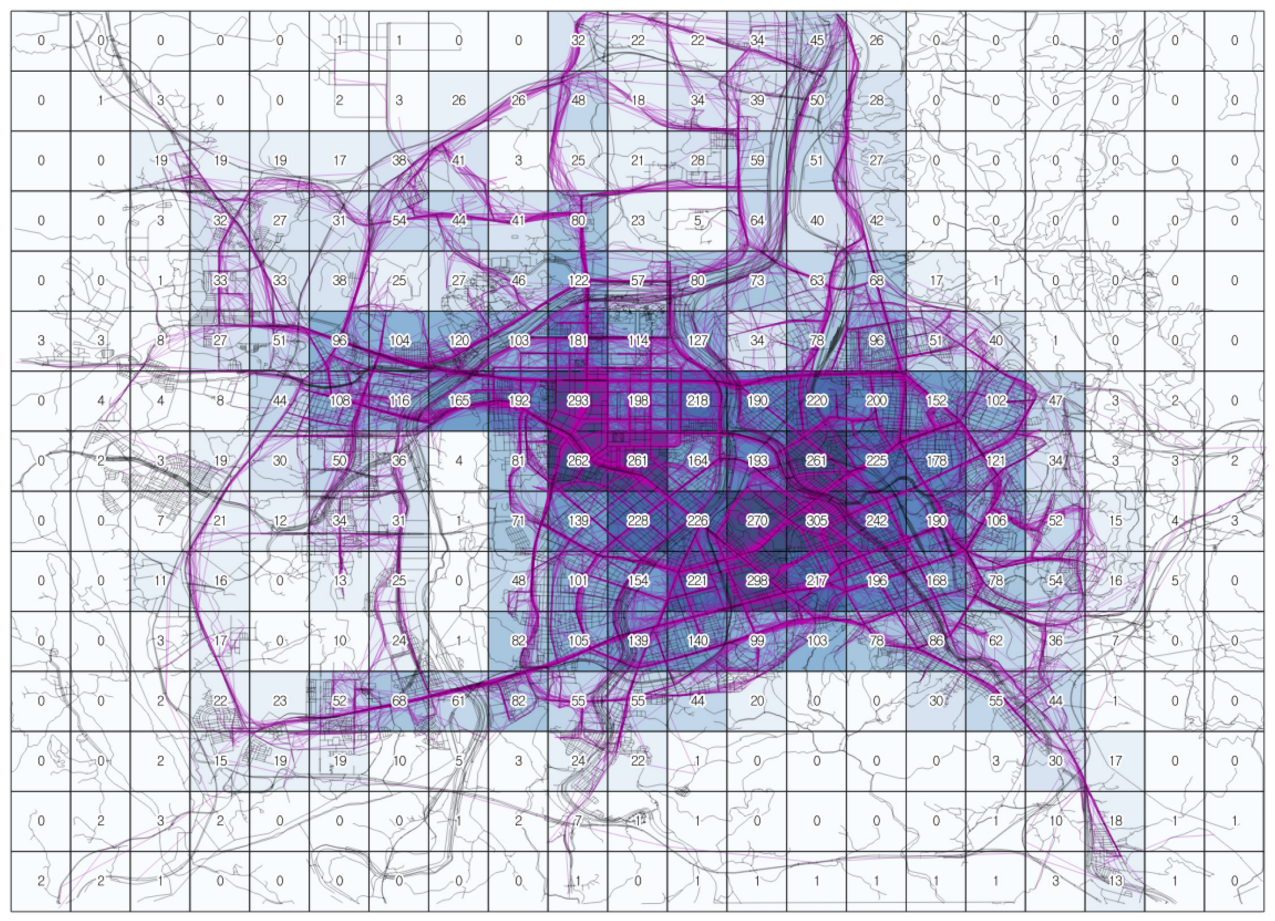
*N*_*veh*_ in each cell.

The optimal CS density for each cell is calculated by the ERDEC model. The parameters *T*_*f_ch*_, $l_{EV_{dis}}$, *L*, *k*, *p* and *T* utilize the default value of the ERDEC baseline model, as shown in Table 3. The parameter *N*_*veh*_ (the number of passing EVs) is the input value that considerably reflects the regional characteristics of each cell. In this instance, *N*_*veh*_ is calculated from the Daejeon taxi data. The Daejeon taxi data include vehicle IDs, event occurrence time, GPS coordinates, and passenger presence; they are recorded every 15 seconds. We select the data from 08:00 to 09:00 in Sep. 23, 2013. Based on the GPS location data, we generate the vehicle trajectory lines using a geographic information system (GIS) and calculate the number of passing vehicles in each cell. The total number of operating vehicles during the study time period is 955. The total number of passing vehicles (lines) in all cells is 12866, whereas the total length of trips in all cells is 13133.16 km. The number of passing vehicles in each cell ranges from 0 to 305, and the average number of passing vehicles is 40.84. Fig 13 shows the passing trajectories of taxi data for the cells in the Daejeon area. The recommended parameters are listed in Table 5.

**Table 5:** Recommended parameters.

Parameter		Description	Value
Technological	*T*_*f_ch*_	*full charging time*	1 hr
	$l_{EV_{dis}}$	*available EV distance*	143.5 km
Regional	*L*	*one side length of the area*	1 km
	*k*	*the number of chargers at station*	1
	*T*	*time state*	1
	*N*_*veh*_	*the number of passing EV*	0 to 305
	*p*	*proportion of peak time demand*	0.6

**Fig 14:**
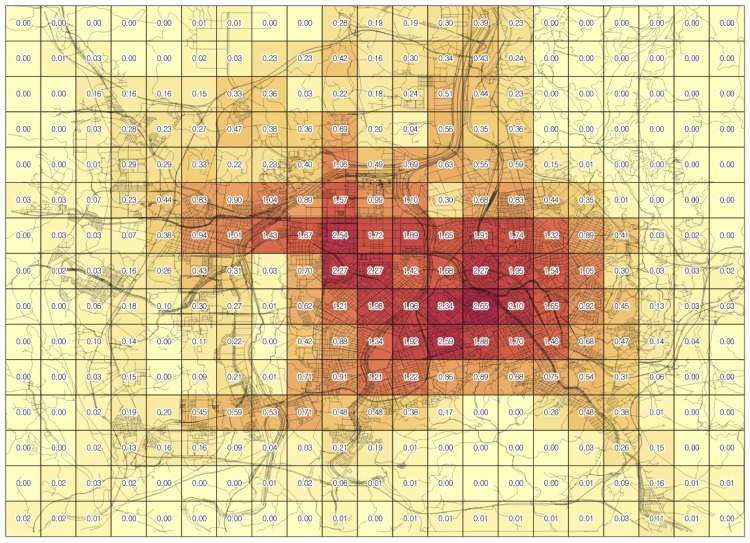
The results map of the optimal density.

In this case, the coefficient factors for the EV taxis (Table 4) are employed because the *N*_*veh*_ value is calculated from the taxi data. With the scenario setting parameters, the subject of this application example is to generate the optimal density map of CSs in Daejeon for 955 of the operating EV taxis without driving-range anxiety. Fig 14 and Table 6 show the results. The average optimal density is 0.35, the median is 0.09, and the optimal density ranges from 0 to 2.65. The sum of the density values is 111.8. The sum of the density values indicates the total number of charging stations. In this case, the number of total charging stations is equivalent to the number of total chargers. The number of charging stations may not seem to be sufficient because *k* is 1. In addition, the number of charging stations for EV is inevitably large to achieve the efficiency of a general gas station because the charging time is much longer than the fueling time (the charging time is approximately 60 minutes, whereas the fueling time is approximately 5 minutes) and the frequency of charging is significant due to the relatively short driving range. After an examination of the results map, the unit areas with relatively high values (more than 2.0) are located near the downtown and public transit stations. This finding provides an approximate solution for distributing the CSs in the economic planning phase. Based on this initial result, the decision makers who install CSs can develop detailed plans for locating a CS within a cell or a group of cells and integrate neighboring cells with low density values.

**Table 6:** The results table.

Basic statistics	Value
Average optimal density	0.3549
Sum of optimal density values	111.8
Minimum optimal density	0
Maximum optimal density	2.65
Median optimal density	0.09
Number of cells	315
Total number of vehicles (lines) in all cells	12866
Total number of CSs	111.8
Total cost	1384112

**Fig 15:**
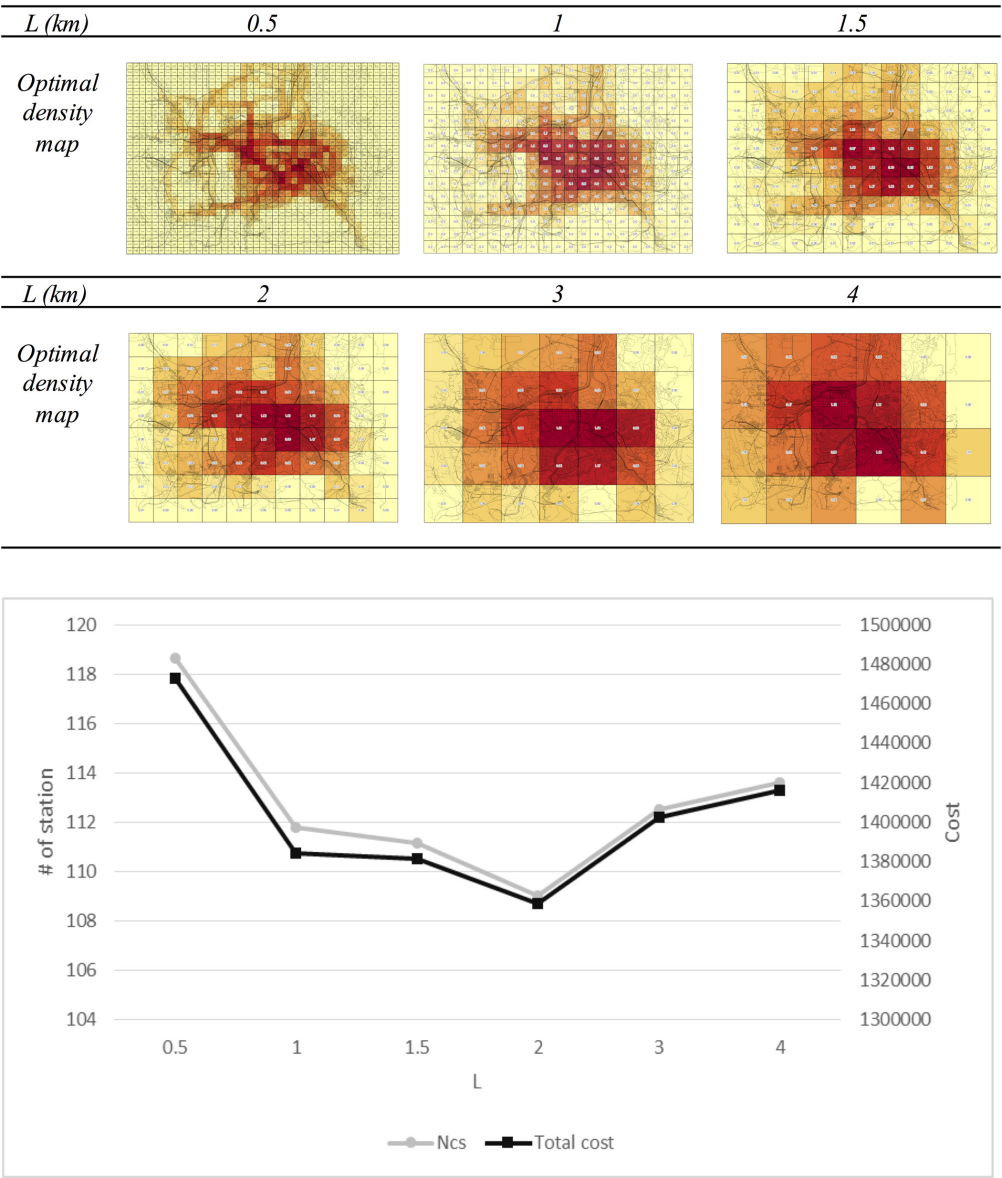
The results according to L size.

To determine the change in the results according to *L* size, the results maps with different *L* sizes are generated as illustrated in Fig 15. The chart shows the minimum value when L is 2 km. In the case in which *L* is 2, the total number of CSs is 109.04 and the total cost is 1358631.

In terms of minimizing the total cost of all cells, the results map for an *L* size of 2 km as the unit of analysis is suitable in the Daejeon case. If *L* is longer, the selection of an exact candidate location of CSs at the next detailed deployment planning phase is uncertain because the size of the unit cell increases. Thus, decision makers should select an appropriate results map after identifying the advantages and disadvantages.

## Conclusions

In the initial stage of introducing EVs, adequate installation of charging infrastructure is crucial for supporting the fast and timely transition to a low-emission society with EVs. To support the determination of CS allocation without wasting public resources, an optimal model for minimizing total cost and matching charging demands is needed. For the same number of vehicles (EV and internal combustion engine vehicles (ICEVs)), the charging stations for EVs are more critical than gas stations for ICEV because EVs have a greater frequency of charging due to the short driving range and because the charging time is much longer than the fueling time. Thus, the efficient deployment plan of charging infrastructure is critical. This paper suggests the ERDEC model as a solution to this problem. This model provide an analytical solution for estimating the optimal density of CSs in the planning phase. The cost of the optimal solution is lower than the total cost of the solution of matching the charging demands while preventing the failure of charging. With this model, we can determine the optimal density of a single unit area (cell) and generate the optimal density map of urban area divided by several cells.

In this paper, based on the ERDEC model, a numerical study is performed with a case study. Daejeon in South Korea is selected as the study site for applying the ERDEC model to the real world. The numerical study analyzes the correlations among the parameters of the ERDEC model, such as regional parameters, technological parameters and coefficient factors. With real taxi trajectory data and the scenario in which 955 EV taxies are operational, the optimal density maps of CSs are generated using the ERDEC model. Decision makers for the installation of CSs in local governments can develop a detailed deployment plan by considering these results.

This study has some limitations; the different conditions in the real world are not satisfied because the ERDEC is a simplified model. First, this study assumes that the vehicles move in a vertical or horizontal direction. Because the predominant layouts of the road networks in urban areas exhibit grid shapes, the proposed method is suitable for urban areas. However, a more advanced method is needed to examine suburban areas that have grid-type road networks. Because the data in the case study are collected from taxis, the results in this study are dedicated to EV taxis. For general passenger EVs, the same type of data on general vehicles in each cell is needed. Future studies should analyze other aspects, such as the comparison of the density result maps of different dates and time periods.

The proposed model can be applied to a relatively extensive range of area to encourage the use of EVs by reducing the range anxiety in the planning phase. It is more applicable to areas with a lack of information, such as exact candidate sites for CSs, travel patterns and other EV-related data. This model can provide an approximate solution with minimum information, such as the number of vehicles per unit area. Another contribution of this model is the prediction of the benefit of reducing the optimal density and total cost by technological developments related to EVs, via various combinations of technological parameters.
